# Advances in Skin Ultrasonography for Malignant and Benign Tumors of the Head and Neck: Current Insights and Future Directions

**DOI:** 10.3390/jcm14072298

**Published:** 2025-03-27

**Authors:** Katarzyna Stawarz, Adam Galazka, Magdalena Misiak-Galazka, Monika Durzynska, Anna Gorzelnik, Karolina Bienkowska-Pluta, Jacek Korzon, Filip Kissin, Jakub Zwolinski

**Affiliations:** 1 Head and Neck Cancer Department, Maria Sklodowska-Curie National Research Institute of Oncology, 02-781 Warszawa, Poland; 2 Department of Pathology, Maria Sklodowska-Curie National Research Institute of Oncology, 02-781 Warszawa, Poland

**Keywords:** high-frequency ultrasonography, basal cell carcinoma, squamous cell carcinoma, melanoma, Mohs surgery

## Abstract

Ultrasound imaging has become an indispensable diagnostic tool across various medical fields. In recent years, there has been growing interest in the use of ultrasonography for the evaluation of skin lesions. However, scientific reports detailing the precise role of ultrasound in determining the morphology of malignant skin tumors still remain limited. Malignant skin lesions, particularly in the head and neck region—their most common location—pose significant challenges due to the complex anatomy of these areas. The primary treatment for non-melanoma skin cancers, including basal cell carcinoma (BCC) and squamous cell carcinoma (SCC), is surgical excision. Mohs micrographic surgery is considered the gold standard due to its tissue-sparing approach and high cure rates. However, it is a time-consuming and resource-intensive procedure that is not always widely accessible. In contrast, standard surgical excision, while more widely available, often results in incomplete tumor removal, necessitating subsequent surgical radicalization or the use of adjuvant therapies. Routine ultrasound evaluation of both benign and malignant skin lesions could enhance early detection and facilitate timely treatment. However, the current body of evidence for the usage of skin ultrasound in presurgical evaluation is poor and lacks standardization. Given these challenges, in this review, we aim to highlight the potential value of preoperative skin ultrasonography in accurately assessing benign and malignant skin lesion dimensions and morphology.

## 1. Introduction

**Figure 1:**
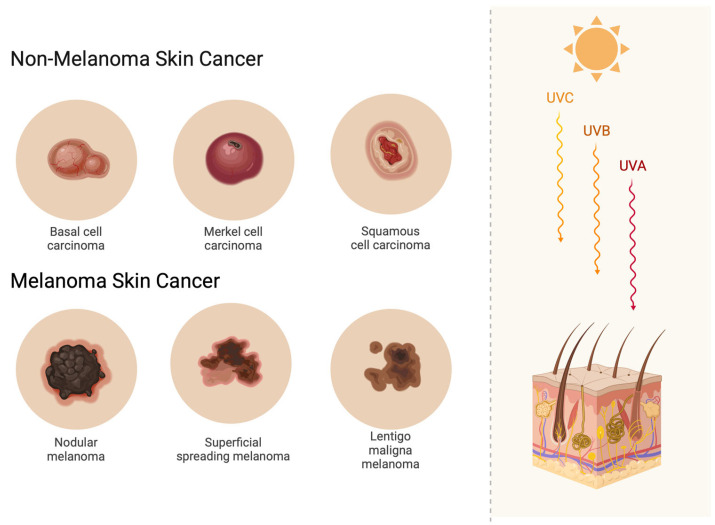
An image illustrating non-melanoma and melanoma skin cancer types, highlighting the correlation between their etiology and sun exposure.

Skin cancer, including basal cell carcinoma (BCC) and squamous cell carcinoma (SCC), remains one of the most prevalent malignancies worldwide [1]. Due to its well-established association with ultraviolet (UV) radiation and sun exposure, it most commonly develops in the head and neck region [2,3] (Figure 1). Moreover, melanoma, which shares a similar etiology, is currently increasing in incidence, with a growing number of new cases reported worldwide [4]. Surgery remains the gold standard for treating skin cancer when the lesion is surgically accessible; however, it may present significant challenges when the lesion is located in the head and neck region [5]. Although treatment strategies for skin cancer management have been well established for many years, presurgical assessment and surgical planning remain challenging processes. While current imaging techniques for skin cancer evaluation, such as dermatologic assessment with dermoscopy and confocal microscopy, can aid in diagnosis, they do not allow for thorough, in-depth evaluation of the lesion [6]. On the other hand, the use of skin ultrasound in the presurgical evaluation of skin cancer has the potential to serve as a valuable tool for cancer diagnosis and surgical planning. However, its application remains limited, particularly in patients with recurrent skin cancer or those who have undergone radiotherapy.

Nevertheless, the implementation of skin ultrasonography offers a valuable tool for presurgical lesion assessment, providing several advantages such as wide availability, noninvasiveness, and cost-effectiveness [7]. Skin ultrasound can be used to accurately measure lesion dimensions during surgical planning and to monitor for potential recurrence [8]. Moreover, it provides a comprehensive, panoramic view of lesion morphology, aiding in the evaluation of tumor margins and surrounding tissue involvement [8].

Despite its benefits, evidence supporting the routine use of skin ultrasound in managing malignant skin lesions remains limited. In the case of melanoma, a biologically diverse malignancy with a wide range of appearances, establishing standardized ultrasonographic criteria for diagnosis is particularly challenging [9]. Melanomas often lack distinct ultrasonographic features that reliably differentiate them from benign pigmented lesions, making dermoscopy the superior method for melanoma diagnosis and assessment [10]. Additionally, even high-frequency ultrasonography (HFUS) cannot reliably detect the subtle pigmentation changes or cellular atypia characteristic of melanoma. Nevertheless, HFUS has the potential to serve as a valuable diagnostic tool for the evaluation of other cancer types, particularly BCC and SCC. 

The objective of this review is to summarize the current state of the literature on the application of benign and malignant skin tumor ultrasound with a particular emphasis on its role in cancer diagnosis, treatment planning, and disease monitoring.

### 1.1. Skin Ultrasound Overview

**Figure 2:**
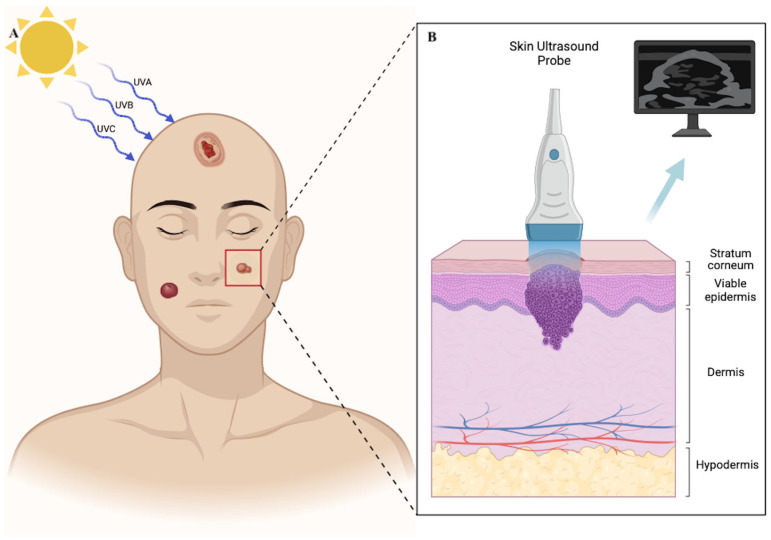
An image depicting the etiology of skin cancer (**A**) and a schematic graph illustrating skin histology, which can be assessed using ultrasound (**B**).

Skin ultrasound imaging serves as a noninvasive technique that uses the physical properties of ultrasound to evaluate both the skin and its appendages [11]. A detailed ultrasonographic assessment can be conducted using high-frequency and very high-frequency probes, ensuring optimal depth penetration and resolution [12] (Figure 2). Although optional, the use of color Doppler can be beneficial for assessing vasculature, aiding in the differentiation between inflammatory processes and tumor neoangiogenesis [13]. Skin ultrasonography can be a valuable tool for evaluating benign cutaneous lesions, malignant tumors, vascular malformations, inflammatory conditions, and overall skin health [14]. Furthermore, among current radiological imaging modalities, ultrasound provides the highest axial spatial resolution, ranging from 100 μm to 30 μm, enabled by advancements in high-frequency technology reaching up to 100 MHz [15]. High-frequency ultrasound (HFUS) typically operates within a frequency range of 20 to 100 MHz. The resolution of high- or ultra-high-frequency ultrasound is significantly greater compared to MRI or PET scans [16]. As a result, small lesions, such as those under 3 mm in MRI or under 8 mm in PET scans, may go undetected [17]. While optical coherence tomography (OCT) offers superior axial spatial resolution compared to ultrasound (approximately 10 μm), its limited penetration depth—reaching only up to 2 mm—restricts its utility in deeper tissue evaluation [18]. The ability to detect blood flow in real time using color or power Doppler makes ultrasound superior to other imaging modalities [19]. Additionally, ultrasound’s capacity to penetrate tissues without significant loss of resolution is particularly crucial for evaluating skin cancer, where assessing deep layer infiltration is of primary importance. The described features of skin ultrasonography are particularly significant in evaluating facial skin lesions, as facial skin is much thinner than skin on other parts of the body [20]. Although Mohs surgery is considered the gold standard for treating BCC, it is a time-consuming procedure and not widely accessible [21]. Additionally, recurrence rates following Mohs surgery are reported in 2–5% of cases [22]. Preoperative ultrasonographic assessment of the lesion can aid in the surgical decision-making process, helping to determine whether Mohs surgery or standard excision is the most appropriate approach. The ability of ultrasound to assess potential cancer infiltration into deeper skin layers, subdermal tissues, including cartilage or bone, makes it a valuable tool in surgical planning [23]. Skin ultrasound can detect not only the primary tumor but also satellite lesions (<2 cm from the primary tumor), in-transit metastases (>2 cm from the primary tumor), and potential nodal metastases [24]. In this review, we aim to analyze the role of skin ultrasonography in evaluating benign and malignant skin tumors located on the facial skin. The illustrative cases presented in this review were conducted using a linear probe with a frequency of 48 MHz (Draminski DermaMed Company, Szabruk, Poland). The figures included in this review are sourced from patients who provided informed consent for the publication of their images. All examinations were performed in compliance with the ethical principles outlined in the Declaration of Helsinki. Additionally, a scoping literature review was conducted in accordance with the PRISMA-ScR 2018 guidelines. The reviewed studies included both research and review articles on skin ultrasound, published between 2010 and 2025, and retrieved from the PubMed and Cochrane databases. The synthesized information was structured into narrative summaries, supplemented by tables and visual representations to enhance clarity.

### 1.2. Normal Facial Skin Ultrasound: Characteristics and Potential Applications

**Figure 3:**
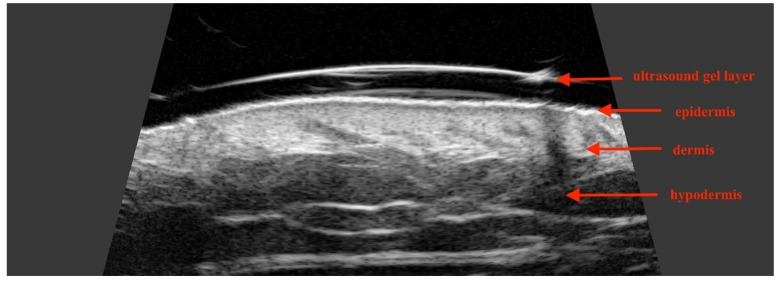
An ultrasound image illustrating skin histology as visualized using high-frequency ultrasound.

To accurately evaluate the skin layers, including the epidermis, dermis, and hypodermis (Figure 3), higher ultrasonographic frequencies are required due to the small dimensions of these structures. A study performed by Wang et al. involving 4338 skin lesions found that integrating ultrasound with clinical evaluation improved diagnostic accuracy from 73% to 97%, achieving a sensitivity of 99% and specificity of 100% [25].

The thickness of the epidermis on the facial surface ranges from 0.04 mm to 0.12 mm, while the dermis varies between 1.0 mm and 4.0 mm, depending on the specific location [26]. The thinnest area is around the eyelids (approximately 1.0 mm), while the thicker regions include the cheeks and forehead, where the dermis can reach up to 3.0–4.0 mm [26,27]. In skin ultrasonography, penetration depth is of paramount importance and is strongly influenced by the frequency of the ultrasound probe. For example, a 22 MHz ultrasound wave can penetrate to a depth of approximately 8–10 mm, a 33 MHz wave to 6–8 mm, and a 75 MHz wave to 3–4 mm [28] (Table 1). HFUS offers exceptionally high resolution, ranging from 80 to 16 μm (micrometers), allowing for the visualization of objects as small as 0.08 mm to 0.016 mm [28]. The hyperechogenicity of the epidermis is attributed to elements with the highest acoustic impedance heterogeneity, including the keratinized scales of the stratum corneum, lipids in the intercellular spaces, and cells in the spinous and basal layers [29]. On the other hand, the thin layer of sub-epidermis is characteristic for low echogenicity and is therefore known as sub-epidermal non-echogenic band (SENEB) [30]. The dermis may exhibit hyperechogenicity, although to a lesser extent than the epidermis, primarily due to its high content of collagen and elastic fibers, as well as nerve fibers and blood vessels [31]. On the other hand, the subcutis layer is predominantly hypoechogenic due to the presence of fat lobules, which allow for ultrasound waves to pass through with minimal impedance [32]. The potential applications of HFUS in assessing skin conditions, both normal and pathological, are detailed in Table 2.

Another important aspect that should be addressed is the standardization of protocols in skin ultrasonography, which is essential for ensuring consistency, accuracy, and reproducibility in dermatologic imaging [33]. One of the fundamental aspects of these protocols is the transducer frequency, which should typically be ≥15 MHz to provide high-resolution imaging of superficial skin structures [14]. The use of ultrasound systems capable of producing high-resolution images is crucial for the accurate assessment of skin lesions, allowing for precise measurements and characterization [14]. In terms of scanning protocols, it is recommended that scans be performed in at least two perpendicular planes (horizontal and vertical) to comprehensively evaluate lesion dimensions, depth, and structural characteristics [16]. Additionally, color Doppler ultrasound should be incorporated to assess vascularity, aiding in the differentiation between benign and malignant tumors by identifying abnormal blood flow patterns [34]. Another critical component of standardization is operator training, as the accuracy and reliability of ultrasonographic evaluations are highly dependent on the clinician’s expertise. To ensure proficiency in image acquisition and interpretation, it is recommended that physicians perform a minimum of 300 skin ultrasound examinations annually [35]. Adhering to these standardized protocols enhances diagnostic accuracy, reproducibility, and clinical utility, ultimately improving patient outcomes in dermatologic ultrasonography.

## 2. Basal Cell Carcinoma

**Figure 4:**
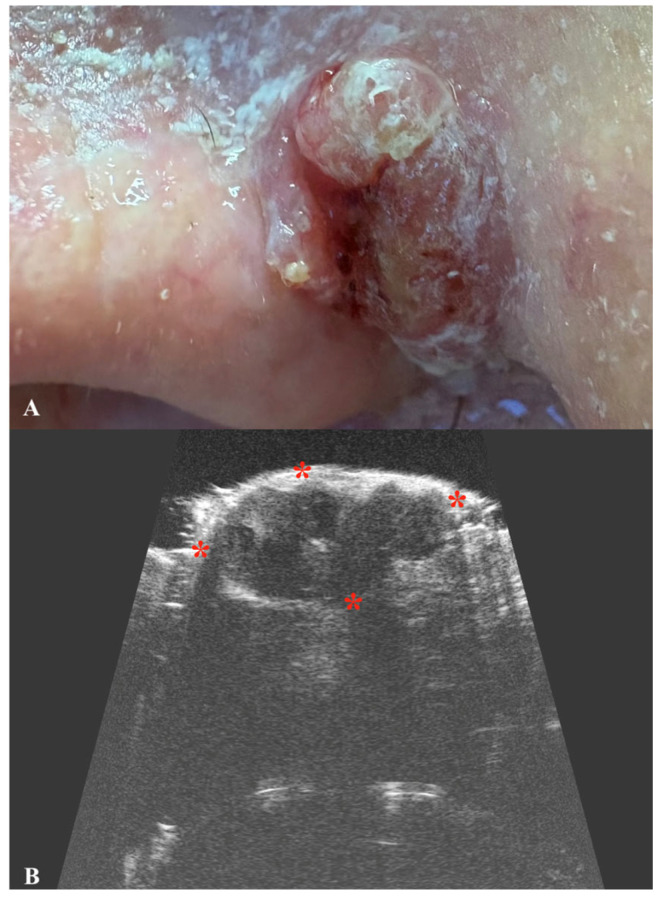
Macroscopic view of basal cell carcinoma (**A**) with a corresponding ultrasound image (**B**), presenting a hypoechoic nodule with a hyperechoic border and multiple intranodular hyperechoic dots. Stars indicate tumor margins.

BCC is the most common skin cancer worldwide, with the number of cases increasing annually [36]. Although it is rarely fatal, BCC may lead to significant local tissue destruction and disfigurement if treatment is delayed or inadequate [37]. Primarily caused by ultraviolet radiation and sun exposure, BCC most commonly occurs on the face, particularly in areas such as the nose, cheeks, forehead, nasolabial folds, and eyelids [38]. Several types of BCC can be identified, with the nodular type being the most commonly diagnosed [39]. In ultrasonographic imaging, it typically appears as an oval hypoechogenic tumor with well-defined borders [40]. BCC may also appear as a hypoechogenic tumor with a ribbon-like, elongated, or rosary-bead pattern and irregular borders [41,42]. Additionally, the adenoid subtype of BCC may display anechoic lacunar cystic areas on ultrasonography [40]. BCC is usually confined to the dermis; however, in advanced stages, it may extend into deeper layers, including the subcutis [43]. Certain subtypes of BCC, particularly those with an infiltrating growth pattern, may exhibit small hyperechogenic areas within the hypoechogenic tumor. These hyperechogenic regions, often described as a “cotton flower” pattern, are considered a pathognomonic sign of BCC [44]. Although the exact etiology of these artifactual spots is not yet well-studied, the “cotton flower” pattern may be attributed to clusters of apoptotic neoplastic cells within the tumor tissue [44,45]. Furthermore, research suggests that in certain BCC subtypes, including micronodular and morpheaform types, the risk of recurrence is positively correlated with the number of “cotton flower” spots observed [44,45]. A threshold of more than seven hyperechoic spots within the tumor at 15 MHz has been identified as a significant marker for distinguishing high-risk from low-risk recurrence subtypes [44,45,46]. Additionally, these hyperechogenic spots may aid in distinguishing BCC from other malignancies, particularly melanoma or SCC, as their ultrasonographic appearance lacks this characteristic pattern [44,45,46]. Findings from other studies on BCC ultrasonography confirm that this imaging modality may accurately assess the tumor thickness and correlate these results with the BCC histological subtype [42,47]. Preoperative evaluation of the deep margins of BCC has been investigated in studies involving Mohs surgery, demonstrating a sensitivity of 96%, specificity of 84%, and accuracy of 91% in measuring deep tumor margins [48]. Additionally, skin ultrasonography can be valuable not only for assessing primary tumors but also for evaluating recurrent or residual lesions. However, this can be quite challenging, as tissue scarring and inflammatory changes within the residual tumor may complicate the accurate assessment of lesion dimensions (Figure 4).

## 3. Squamous Cell Carcinoma

**Figure 5:**
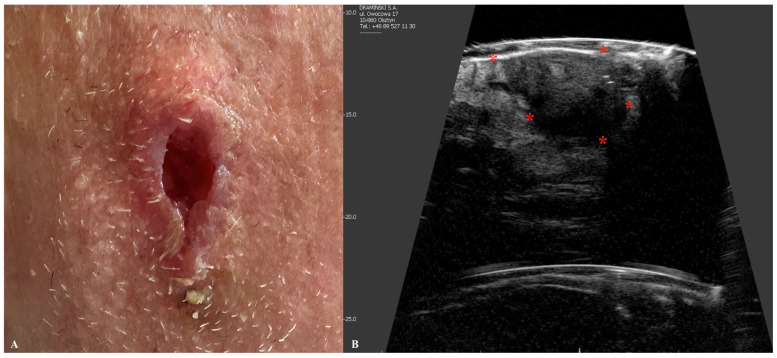
Macroscopic image of SCC (**A**) with a corresponding ultrasonographic image (**B**) showing a hypoechoic infiltrative nodule with irregular, ill-defined borders. Stars indicate tumor margins.

Similarly to BCC, squamous cell carcinoma usually presents as a hypoechogenic lesion within the dermis [49]. On the other hand, SCC is a more aggressive form of skin cancer compared to BCC [50]. HFUS can aid in the assessment of locoregional satellite tumors, which are more frequently associated with SCC [51]. More advanced stages may infiltrate deeper layers including subcutaneous tissues, muscles, cartilages, or bone [52]. The thickened, hyperkeratotic stratum corneum in SCC may hinder precise ultrasonographic evaluation due to the formation of an acoustic shadow artifact, resulting from the reflection and scattering of ultrasound waves [53]. Therefore, although HFUS might be helpful in preoperative assessment of lesion dimensions, the accuracy of this method might not always be satisfactory. For instance, Wortsman et al. demonstrated a modest yet significant improvement in diagnostic accuracy with the use of higher-frequency ultrasound [54]. Another study highlighted the precision of HFUS in measuring SCC depth, showing a remarkable correlation with histometric analysis down to one-hundredth of a millimeter [55]. Although SCC lacks the hyperechogenic dots characteristic of BCC, its ultrasonographic features include epidermal irregularities, a crumpled surface, epidermal detachment, ulceration, and various shapes such as bulging, convex, concave, or flat contours [56,57]. On color Doppler imaging, SCC exhibits higher vascularity compared to BCC, typically characterized by the presence of low-flow vessels [58]. In Bowen’s disease, intradermal involvement is evident, and the ultrasonographic image typically reveals epidermal irregularities, a wavy and crumpled epidermal surface, and a hypoechogenic appearance in the lower portion of the epidermis [59]. Recurrent or residual SCCs exhibit ultrasonographic features similar to those of primary tumors [59]. Scar tissue, characterized by a laminar pattern and tissue heterogeneity, is often observed at the tumor’s periphery. Additionally, varying degrees of vascularity, including the presence of low-flow vessels, can be identified either within the lesion or along its periphery [60] (Figure 5).

## 4. Merkel Cell Carcinoma

**Figure 6:**
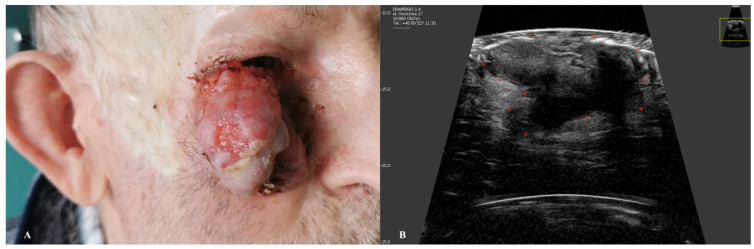
Macroscopic image of Merkel cell carcinoma (**A**) with a corresponding ultrasonographic image (**B**). Stars indicate tumor margins. The yellow box indicates the area covered by the ultrasonographic field of view.

This rare but aggressive and fast-growing tumor originates from Merkel cells [61]. On ultrasound imaging, it appears as a hypoechogenic dermal or hypodermal lesion with ill-defined margins [62]. The vascular pattern is often chaotic [62]. Clinically, the tumor typically presents as an exophytic nodule [63]. The characteristic ultrasound pattern for this malignancy remains poorly understood. However, some studies have highlighted hypoechoic linear bands with a “plume-of-smoke” appearance and various vascular pattern, oriented perpendicularly to the skin surface, observed in some cases of primary Merkel cell carcinoma [64] (Figure 6).

## 5. Melanoma

**Figure 7:**
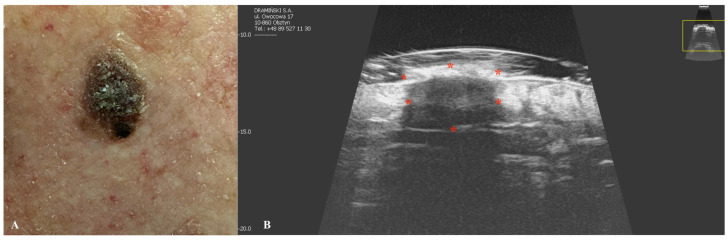
Macroscopic view of malignant melanoma (**A**) with a corresponding ultrasonographic image (**B**). Stars indicate tumor margins. The yellow box indicates the area covered by the ultrasonographic field of view.

Melanoma is a type of skin cancer characterized by one of the lowest survival rates and a rapidly increasing incidence globally [65]. HFUS imaging of melanoma typically reveals hypoechoic, heterogeneous lesions with an oblong or oval shape, often well demarcated by a hyperechoic epidermal layer [66,67]. In cases of ulcerated malignancies, the epidermis may appear discontinuous or irregular [67]. As an angiogenic tumor, melanoma exhibits hypervascularity on color Doppler imaging. Blood flow signals are more readily detectable in lesions exceeding 2 mm in thickness [68]. The degree of vascularization has been correlated with both lymph node involvement and patient survival outcomes [68]. Preoperative HFUS can be valuable in determining surgical margins, as HFUS-measured tumor thickness shows strong correlation with histopathological measurements, particularly in superficial melanomas [69]. However, the accuracy of HFUS is reduced in nodular and vertically growing tumors. The correlation between HFUS and histopathology is most reliable for neoplasms thicker than 2 mm, often allowing for single-stage excision without the need for margin widening [66]. For thinner lesions (<2 mm), either excision or reassessment using a 100 MHz probe is recommended [66]. It should be noted that HFUS measurements may slightly overestimate tumor thickness due to peritumoral inflammation and tissue dehydration following excision [68,70]. Comparative studies have shown varying results regarding imaging modalities. Hinz et al. demonstrated that 1325 nm optical coherence tomography (OCT) provides greater precision than 20 MHz HFUS for melanomas thinner than 1 mm [71]. Conversely, Meyer et al. found that 25 MHz HFUS outperformed 930 nm OCT in assessing tumor thickness, particularly for lesions deeper than 0.5 mm [72]. In postoperative settings, HFUS serves as an effective tool for monitoring regional lymph node basins and detecting satellite or in-transit melanoma metastases [73]. These metastases typically present as well-defined, hypoechoic, relatively homogeneous structures in the dermal or hypodermal layers [73] (Figure 7).

## 6. Mycosis Fungoides

**Figure 8:**
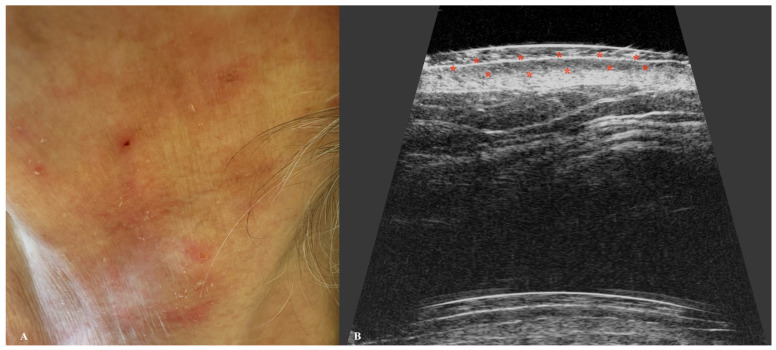
Macroscopic view of early stage mycosis fungicides (**A**) with a corresponding ultrasonographic image (**B**). Stars indicate tumor margins.

Another malignancy within the non-melanoma skin cancer group that can be assessed using HFUS is mycosis fungoides (MF). MF is characterized by the presence of a SLEB, which has been identified as a useful marker for evaluating therapeutic response and disease remission [30]. In two independent studies involving MF patients, thinning or complete disappearance of the SLEB was correlated with disease severity and successful completion of therapy [74,75]. These findings highlight the potential utility of HFUS as an objective tool in routine clinical practice (Figure 8).

## 7. Fibroma

**Figure 9:**
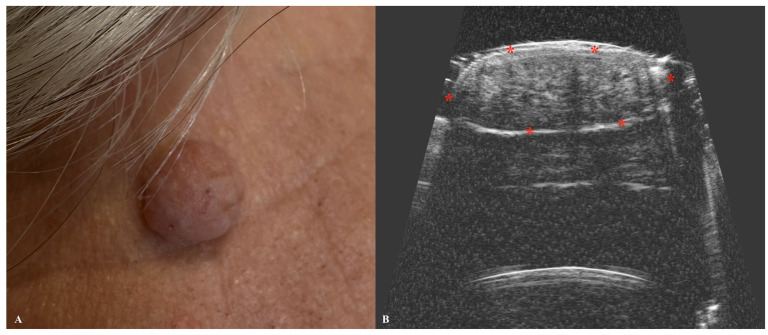
Macroscopic view of a forehead fibroma (**A**) with a corresponding ultrasonographic image (**B**), showing a well-defined tumor with mixed echogenicity and a hyperechoic surrounding band. Stars indicate tumor margins.

This benign cutaneous tumor, composed of connective tissue, can develop on any part of the body, including the face [76]. On HFUS, it appears as a well-defined, hypoechoic epidermal lesion located just above the dermis. Additionally, these tumors lack any detectable vascular pattern on color Doppler imaging [77]. In rare cases of neoplastic degeneration, changes in the sonographic pattern may be observed, including an inhomogeneous structure and ill-defined margins [77] (Figure 9).

## 8. Epidermoid Cyst

**Figure 10:**
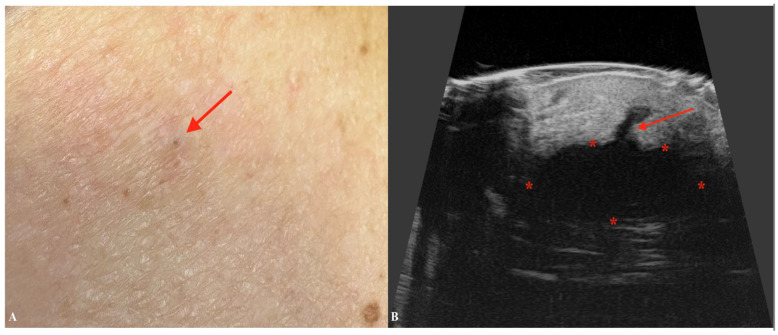
Macroscopic image of a cheek epidermoid cyst (**A**), with an arrow indicating the cyst orifice. The corresponding ultrasound image (**B**) displays the epidermoid cyst with a visible duct (arrow). Stars indicate the lesion margins.

This benign lesion typically appears as a hypoechogenic mass with well-defined borders [78]. It is generally ovoid or spherical in shape, although it may also present as tubular or lobulated [79]. Larger lesions may exhibit mild heterogeneity due to the presence of mucoid material, fat, calcifications, or pus [78,80]. Typically, no associated vascularity is observed. However, ruptured lesions can display variable characteristics, including occasional vascularity and lobulated contours. On color Doppler, a twinkling artifact may also be detected. Posterior sound enhancement might also be observed [78,80] (Figure 10).

## 9. Dermatofibrosarcoma Protuberans

**Figure 11:**
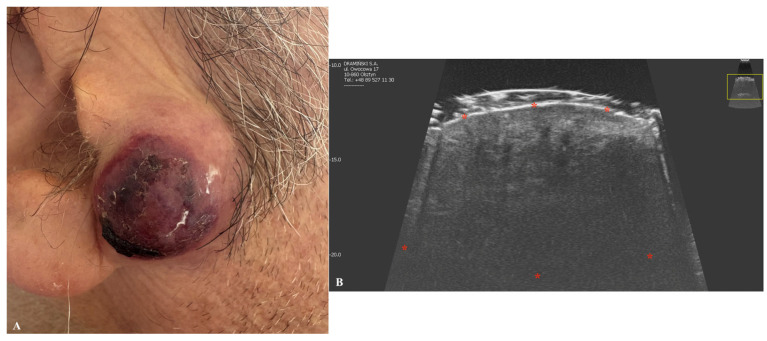
Macroscopic view of dermatofibrosarcoma protuberans (**A**) with a corresponding ultrasound image (**B**). Stars indicate tumor margins. The yellow box indicates the area covered by the ultrasonographic field of view.

This rare skin malignancy exhibits variable morphology on ultrasound imaging, including round or ovoid shapes, lobulated contours, or “tentacle-like projections”, often with irregular borders [81]. The echogenicity also varies, ranging from mixed heterogeneity to hypoechogenicity [82]. In some cases, small echogenic foci may be observed, typically without a comet-tail artifact [83]. Color Doppler imaging reveals low to moderate vascularity, which is generally more prominent at the periphery than in the central region. Acoustic enhancement is also commonly noted [83] (Figure 11).

## 10. Primary Cutaneous Lymphoma

**Figure 12:**
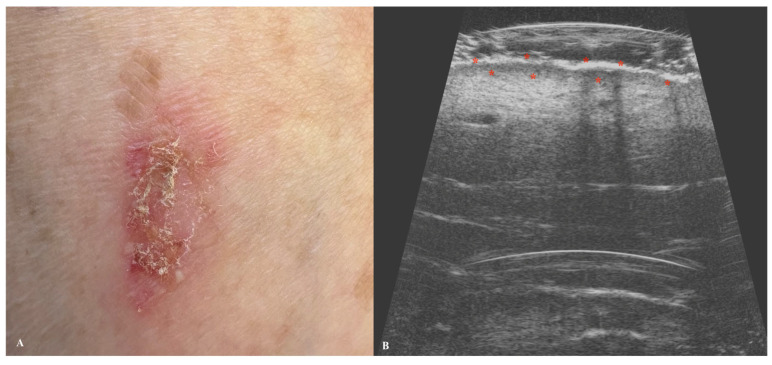
Macroscopic view of cutaneous lymphoma (**A**) with a corresponding ultrasonographic image (**B**). Stars indicate tumor margins.

Primary cutaneous lymphoma (PCL) lesions exhibit various sonographic patterns, including hypoechoic focal infiltrative, nodular, pseudonodular, and diffusely infiltrative appearances [84]. These lesions typically lack internal necrosis or calcifications and demonstrate high vascularity on color Doppler imaging [84]. Focal nodular and pseudonodular lesions are more commonly associated with B-cell lymphomas, while diffusely infiltrative patterns are more frequently observed in T-cell lymphomas [84,85]. HFUS provides detailed visualization of the extent of lesion infiltration within the cutaneous layers and serves as a valuable tool for monitoring the local progression of the disease [86] (Figure 12). A detailed description of all the presented skin tumors along with their ultrasonographic features is provided in Table 3.

## 11. Latest Ultrasonographic Imaging Techniques

Additional advanced imaging techniques that should be highlighted in this review include functional ultrasound imaging (fUS), which has potential applications in brain imaging [87]. This technique is based on ultrasensitive Doppler technology to monitor subtle blood flow variations, facilitating the mapping of brain activity with high spatial and temporal resolution—all without the need for contrast agents [88]. Another recent advancement is elastography, a modality that assesses tissue stiffness by measuring the propagation speed of shear waves induced by acoustic radiation force. This technique is particularly useful in differentiating benign from malignant lesions in organs such as the liver, breast, and thyroid [89,90]. Additionally, three-dimensional (3D) ultrasound, which reconstructs multiple two-dimensional images to create a volumetric representation of anatomical structures, is primarily used in fetal ultrasonography [91]. However, its potential application in skin ultrasound represents an exciting future direction, allowing for more detailed visualization and assessment of dermatologic conditions.

## 12. Clinical Implications and Limitations

Skin ultrasonography is a valuable tool for the early diagnosis of skin tumors and the monitoring of treatment response, aiding in the assessment of therapeutic efficacy. A key advantage of HFUS is that it is a safe and noninvasive imaging technique, enabling real-time evaluation of skin tumors without exposure to ionizing radiation [9]. However, certain limitations must also be considered. While high-frequency probes offer excellent resolution, their penetration depth is limited, making the assessment of deeper skin layers and subcutaneous tissue challenging [92]. Additionally, some benign and malignant lesions may exhibit overlapping ultrasound features, necessitating the use of complementary diagnostic methods, particularly in the case of pigmented lesions, which may further obscure ultrasound findings [10]. Lastly, advanced HFUS devices may not be widely accessible in all clinical settings, particularly in resource-limited regions, restricting their routine use in dermatologic diagnostics.

## 13. Future Perspectives of HFUS

While HFUS already enables detailed skin tumor assessment, further research is necessary to enhance its diagnostic capabilities. The development of higher-frequency probes (>50 MHz) is expected to improve the visualization of fine skin structures at the cellular level, thereby enhancing skin tumor detection [45]. Additionally, three-dimensional (3D) ultrasound imaging could provide volumetric data, improving the assessment of tumor size, shape, and depth, while four-dimensional (4D) imaging could offer additional insights into blood flow dynamics [93]. Future technological advancements should also focus on HFUS with contrast agents, which can enhance the assessment of skin tumor vascularization [94]. This innovation could be particularly valuable in distinguishing aggressive tumors characterized by increased neovascularization. Lastly, in the era of artificial intelligence (AI), the integration of AI algorithms and deep learning models has the potential to revolutionize automatic image analysis, lesion classification, and real-time decision support [95]. AI-powered ultrasound may help reduce operator dependence, improve diagnostic consistency, and minimize human error, ultimately allowing for more precise tumor margin delineation and improved patient outcomes.

## 14. Conclusions

HFUS is a valuable tool in clinical practice, not only for evaluating benign skin conditions but also as an essential modality in the diagnostic assessment of skin oncology. One of the future objectives of HFUS in skin oncology is to refine ultrasonographic protocols to accurately differentiate benign lesions from malignant skin tumors. Moreover, despite its simplicity, HFUS remains a widely accessible, relatively cost-effective, and noninvasive imaging technique that can be used not only for the preoperative evaluation of skin lesions but also for postsurgical surveillance to detect potential tumor recurrence.

## Figures and Tables

**Table 1:** Ultrasound frequency with corresponding penetration depth and potential tissue visualization.

Ultrasound Frequency and Corresponding Tissue Visualization		
Ultrasound Frequency	Depth of Penetration	Visualization
7.5	>4.0	Subcutis and lymph nodes
13.5–50	3.0–0.3	Epidermis and dermis
20	0.6–0.7	Epidermis and dermis
50–100	0.3–0.015	Epidermis

**Table 2:** Potential applications of high-frequency ultrasound (HFUS) in various skin conditions.

Category	Application
Healthy Skin and Aging	Study of micro-anatomical structure of healthy skin and echo-graphic signs of aging.
Cosmetic and Plastic Surgery	Assessment of skin condition and anatomical features before procedures like Botox or fillers injections, post-procedure follow-up including efficacy, side effects, and location of fillers.
Differential Diagnosis and Pathology Management	Support for differential diagnosis and management of various skin pathologies, including presurgical assessment.
Therapeutic Follow-Up	Monitoring response to pharmacotherapy; topical therapy; physiotherapeutic, destructive skin treatments; and potential disease recurrence.
Dermato-Oncology	Assessment of skin tumors characteristics, margins, and vascular patterns in dermato-oncology. Follow-up after surgical tumor removal.

**Table 3:** Detailed description of the ultrasonographic features of the presented skin tumors.

Tumor Type	Ultrasonographic Features
Basal Cell Carcinoma	Oval, hypoechogenic lesion with well-defined borders. Hyperechoic spots (“cotton flower” pattern) may be present. Poor vascularization on Doppler.
Squamous Cell Carcinoma	Hypoechogenic lesion with irregular, poorly defined margins. Epidermal detachment and ulceration are common. More vascularized than BCC.
Merkel Cell Carcinoma	Hypoechogenic dermal or hypodermal lesion with chaotic vascular patterns. May exhibit hypoechoic linear bands (“plume-of-smoke” appearance).
Melanoma	Hypoechoic, heterogeneous lesion with an oblong or oval shape. Often well demarcated by a hyperechoic epidermal layer. Hypervascular on Doppler.
Mycosis Fungoides	Presence of a subepidermal low-echogenic band (SLEB). Useful in monitoring disease progression and response to therapy.
Fibroma	Well-defined, hypoechoic epidermal lesion. Typically avascular on Doppler imaging.
Epidermoid Cyst	Hypoechogenic mass with well-defined borders. May show posterior acoustic enhancement. Occasionally presents mild heterogeneity.
Dermatofibrosarcoma Protuberans	Lobulated hypoechoic lesion with “tentacle-like projections” and moderate vascularity, often more prominent at the periphery.
Primary Cutaneous Lymphoma	Hypoechoic focal infiltrative or nodular lesions with high vascularity. B-cell lymphomas appear more nodular; T-cell lymphomas are more diffuse.

## Data Availability

No new data were created or analyzed in this study. Data sharing is not applicable to this article.

## Abbreviations

3D	three-dimensional
AI	artificial intelligence
BCC	basal cell carcinoma
fUS	functional ultrasound
HFUS	high-frequency ultrasonography
MF	mycosis fungoides
MRI	magnetic resonance imaging
OCT	optical coherence tomography
UV	ultraviolet radiation
PCL	primary cutaneous lymphoma
SCC	squamous cell carcinoma
SLEB	subepidermal low echogenic band
